# Excited-state charge polarization and electronic structure of mixed-cation halide perovskites: the role of mixed inorganic–organic cations in CsFAPbI_3_†

**DOI:** 10.1039/d2ra04513c

**Published:** 2022-09-07

**Authors:** Roghayeh Imani, Carlos H. Borca, Meysam Pazoki, Tomas Edvinsson

**Affiliations:** Department of Materials Science and Engineering, Solid State Physics, Ångström Laboratory, Uppsala University Box 34 Uppsala 75121 Sweden tomas.edvinsson@angstrom.uu.se; Department of Chemical and Biological Engineering, School of Engineering and Applied Science, Princeton University Princeton 08544 New Jersey USA; Institute for Photovoltaics, Stuttgart University Stuttgart 70569 Germany meysam.pazoki@gmail.com; Department of Physics, Shiraz University Shiraz 71454 Iran

## Abstract

Mixed-cation perovskite materials have shown great potential for sunlight harvesting and have surpassed unmixed perovskite materials in solar cell efficiency and stability. The role of mixed monovalent cations in the enhanced optoelectronic properties and excited state response, however, are still elusive from a theoretical perspective. Herein, through time dependent density functional theory calculations of mixed cation perovskites, we report the electronic structure of Cs formamidinium (FA) mixed cationic lead iodide (Cs_0.17_FA_0.87_PbI_3_) in comparison to the corresponding single monovalent cation hybrid perovskite. The results show that the Cs_0.17_FA_0.87_PbI_3_ and FAPbI_3_ had negligible differences in the optical band gap, and partial and total density of states in comparison to a single cation perovskite, while the effective mass of carriers, the local atomic density of states, the directional transport, and the structural distortions were significantly different. A lattice-distortion-induced asymmetry in the ground-state charge density is found, and originates from the co-location of caesium atoms in the lattice and signifies the effect on the charge density upon cation mixing and corresponding symmetry breaking. The excited-state charge response and induced polarizabilities are quantified, and discussed in terms of their importance for effective light absorption, charge separation, and final solar cell performance. We also quantify the impact of such polarizabilities on the dynamics of the structure of the perovskites and the implications this has for hot carrier cooling. The results shed light on the mechanism and origin of the enhanced performance in mixed-cation perovskite-based devices and their merits in comparison to single cation perovskites.

## Abstract 2

The roles of mixed monovalent cations in CsFAPbI_3_ perovskites in terms of the optoelectronic properties and excited-state charge polarization are reported.

## Graphical abstract

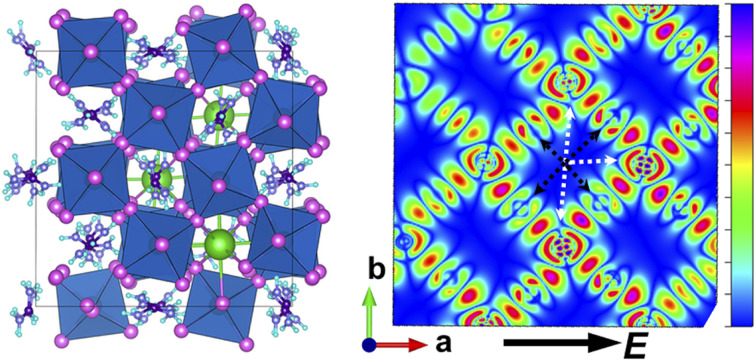

## Introduction

The emergence of effective perovskite solar cells (PSCs)^1–3^ has attracted extraordinary interest among researchers towards hybrid lead halide perovskites and has led to state-of-the-art solar cell devices with power conversion efficiencies approaching record Si-solar cell efficiencies. The main member of the PSC materials family is methylammonium lead iodide (MAPbI_3_), but it is volatile and unstable in UV and blue light. Formamidinium lead iodide (FAPbI_3_), and caesium lead iodide (CsPbI_3_) have instead a higher stability using low wavelength light, and less thermal degradation compared to MAPbI_3_. These compounds, however, also suffer from limited photoactive phase stability at room temperature. In the last few years, compositional engineering has developed different perovskite materials, with improved stability and efficiency. Notably, organic–inorganic mixed-cation and mixed-halide perovskite compounds have been developed with several performance and stability advantages in comparison to single monovalent cation perovskite compounds.^4–7^ Currently, the cutting-edge perovskite materials for solar cell applications, in terms of photovoltaic performance, light/heat/moisture stability, and band gap tunability for tandem applications, are mixed A-site cation perovskites. The main benefits of mixed-cation perovskites are the possibility of engineering new perovskite compounds with higher stability, better solar cell performance, and suitable band gap for tandem applications.^4^ Here, the so-called inverted structure is necessary to enable stacking with another solar cell material. So far, the highest efficiency reported for single junction solar cells based on mixed-cation perovskites in the inverted device structure is 25%.^8^ However, a higher efficiency of 31.25% and a one-thousand-hours stability test under illumination in damp and hot conditions has been reported for tandem solar cells based on mixed-cation perovskites and silicon.^9^ The intrinsic reason and theoretical background of the improved efficiency and stability by mixing of A-site cations in the lead halide perovskites for both normal (PCE_record_ = 25.7%, certified) and inverted structure (PCE_record_ = 25.0%, uncertified) are until now less studied. Theoretical studies of mixed-cation perovskite materials can here provide the origin and rationale of the benefits of a mixed cation system. Physical properties of MAPbI_3_ have previously been investigated by means of first-principles calculations in many studies as highlighted in a recent book.^10^ Unlike the case of MAPI_3_, however, the electronic structure of mixed-cation and mixed-anion lead perovskites has not been widely investigated. The presence of mixed cations in the lattice can affect their electronic and photophysical properties, such as the selection rules of the light absorption or ionic movement. The main electronic states which are responsible for photovoltaic operation come from the inorganic network, and the presence of monovalent cation can indirectly affect the light absorption and charge-transport phenomena by tilting of the inorganic octahedra. Brauer *et al.* through a combination of transient absorption, photoluminescence experiments, and density functional theory (DFT) calculations, suggested that less symmetry of lattice allows the involvement of more high-energy states in the charge excitation and relaxation, therefore leading to longer electron and hole lifetimes and slower hot electron cooling.^11^ Szemjonov *et al.*, calculated the effect of oxygen replacement in the iodine vacancy for the mixed perovskite FA_0.85_MA_0.11_Cs_0.04_PbI_3_ and concluded that oxygen replacement increases the stability of mixed perovskites.^12^

A higher performance,^5^ longer excited-state lifetime,^11,13^ less defect states,^14^ less current voltage hysteresis,^13^ and better stability^5,6^ of mixed-cation perovskites can stem from diverse factors; for instance, from a lower degree of ionic movement.^15^ One of the main sources for instability of perovskite devices, both in material composition and delivery of power, is the ionic movement where the defects can migrate to and along grain interfaces and change the electronic states; triggering degradation mechanisms at the interface.^16^ The barriers of ionic movement in perovskite materials depend on the lattice symmetries and the type of monovalent-cation. In this regard, we previously reported on the monovalent cation roles in phonon-assisted ionic movement,^17^ interactions with vacancies,^18^ and dielectric relaxation^19^ in single monovalent cation perovskites. Additionally, the lattice asymmetry modifies the electronic structure of mixed-cation perovskites compared to single-cation perovskites. These differences can explain the performance dissimilarities of mixed-cation and single-cation perovskites for example in ionic movement or the dielectric constant of the material. Light absorption includes a series of electronic transition from the valence band to conduction band that is affected by selection rules coming from symmetries of charge density in ground state, as reported previously.^20^ However, monovalent cation key-rules are important in mixed-cation perovskites. As the main operation of the material is under continuous illumination, the excited state properties are here vital to include for a full understanding. The role of the mixed cations and, more importantly, how this affects the charge polarization under illumination, is not yet well understood and is one of the main motivations for the subject of this study.

With a band gap of 1.7 eV, Cs_0.17_FA_0.87_PbI_3_ shows a relative high voltage, enhanced performance/stability, low defect density, and represents a mixed-cation perovskite compound with enhanced optoelectronic properties compared with the corresponding single A-site cation perovskites (CsPbI_3_ and FA PbI_3_). Mixed-cation materials with band gaps of 1.7 eV are considered one of the best materials for tandem solar cell fabrication with silicon, and they have even been studied in solar cell concentrator applications under high-intensity illumination.^21^ Herein, we investigate the mixed-cation perovskite Cs_0.17_FA_0.87_PbI_3_ by means of first-principles calculations with the intention to elucidate the origin of the enhanced optoelectronic properties. Electronic structure, geometrical parameters, partial and total density of states, charge-density plots, and excited-state charge-density response are reported and discussed in context of solar cell applications and compared with single monovalent cation perovskites. Insights about the interplay of the mixed monovalent cations in the stability and in the carrier cooling mechanism in mixed perovskites are concluded from the data and discussed. This is useful for both a fundamental understanding of the optoelectronic response and also for designing perovskite films with a minimum ionic movement and highest stability. Furthermore, the results presented in this study can be partially extended to other mixed perovskite materials.

## Computational methods

The electronic structure of the mixed-cation perovskite Cs_0.17_FA_0.87_PbI_3_ was explored *via* density functional theory (DFT) using the Quantum Espresso package.^22^ A large system with 32 unit cells of tetragonal perovskite were included in the implemented structure, 4 of which were occupied with Cs^+^ cations and the rest with FA^+^ cations; the super cell consisted of 356 atoms. Different orientations of dipolar cations were considered in order to obtain the structure with minimum energy as mentioned in our previous reports.^18,19,23,24^ Four Cs monovalent cations were placed in different locations of the lattice; for each configuration, variable cell relaxation, atomic coordinates relaxation and self-consistent calculations were performed and the configuration with minimum energy was finally selected for further calculations.

Initial lattice parameters of the single tetragonal unit cell were *a* = 8.71 Å, and *c* = 12.46 Å.^18^ Scalar relativistic calculations with a pseudo-potential approximation and the PBE functional were employed to obtain the electronic structure, partial density of states, and charge density. The PBE functional has been reported to capture the experimental band gaps well for the lead iodide perovskites. Here an error cancellation occurs causing the DFT bandgap to approximate the exact experimental value as mentioned previously.^25^ A variable cell relaxation was carried out at the *Γ* point while the rest of calculations were performed with 2 × 2 × 2 lattice points in a Monkhorst grid. The lead 5d^10^/6s^2^/6p^2^, the nitrogen 2s^2^/2p^3^, the iodide 5s^2^/5p^5^, and the carbon 2s^2^/2p^2^ electrons were considered as valence electrons. For ground states calculations, the Kohn–Sham orbitals were expanded in a planewave basis set with cut off energies for the electronic wave and charge density of 30 and 350 Rydberg, respectively, after convergence tests. Self-consistency in the total energy was achieved with a tolerance of 10^−5^ Rydberg. The relaxation procedure regarding the unit cell vectors and atomic coordinates continued until the forces acting on atoms were less than 0.06 Ry per a.u. The VESTA^26^ and XCrysDen^27^ packages were used for visualization of unit cell and related charge densities.

Time dependent density functional theory (TD-DFT) calculations were performed using the Turbo TD-DFT code^28^ in the Quantum Espresso package. The linear charge-density response of Cs_0.17_FA_0.87_PbI_3_ was estimated from the incoming light (here polarized in different directions) based on the Liouville–Lanczos approach. 5000 iterations were implemented to calculate the charge-density response for charge excitations from polarized incoming light (monochromic light polarized along *z* axes or lattice *ab*-plane, photon energy = 395 nm). The cut off energy for TD-DFT calculations was set to 70 Ry. The charge-density response is calculated as the solution of the linearized Liouville equation:^28^1
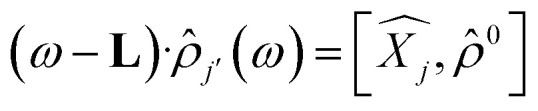
2
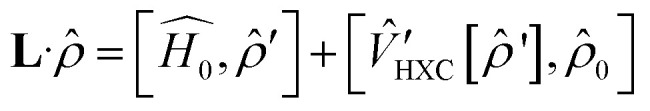
where *ω* is the frequency of incoming light with polarization along the *j*-direction, *ρ̂* and *ρ̂*′ are charge-density responses, and the ground-state charge-density, respectively. 
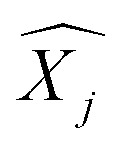
 is the *j* component of the dipole moment operator and **L** is the time-dependent quantum Liouvillian operator defined by eqn (2). Here 
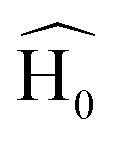
 and 
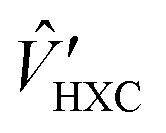
 are the ground-state Hamiltonian and the incoming light induced linear correction, respectively, while more details about this approach have been reported previously.^28,29^ There is no possibility to relax the atoms during or after the excitations in Quantum Espresso, and the presented data are corresponding to the moments immediately after excitation and before lattice relaxation and carrier cooling.

## Results

### Structural properties

**Fig. 1:**
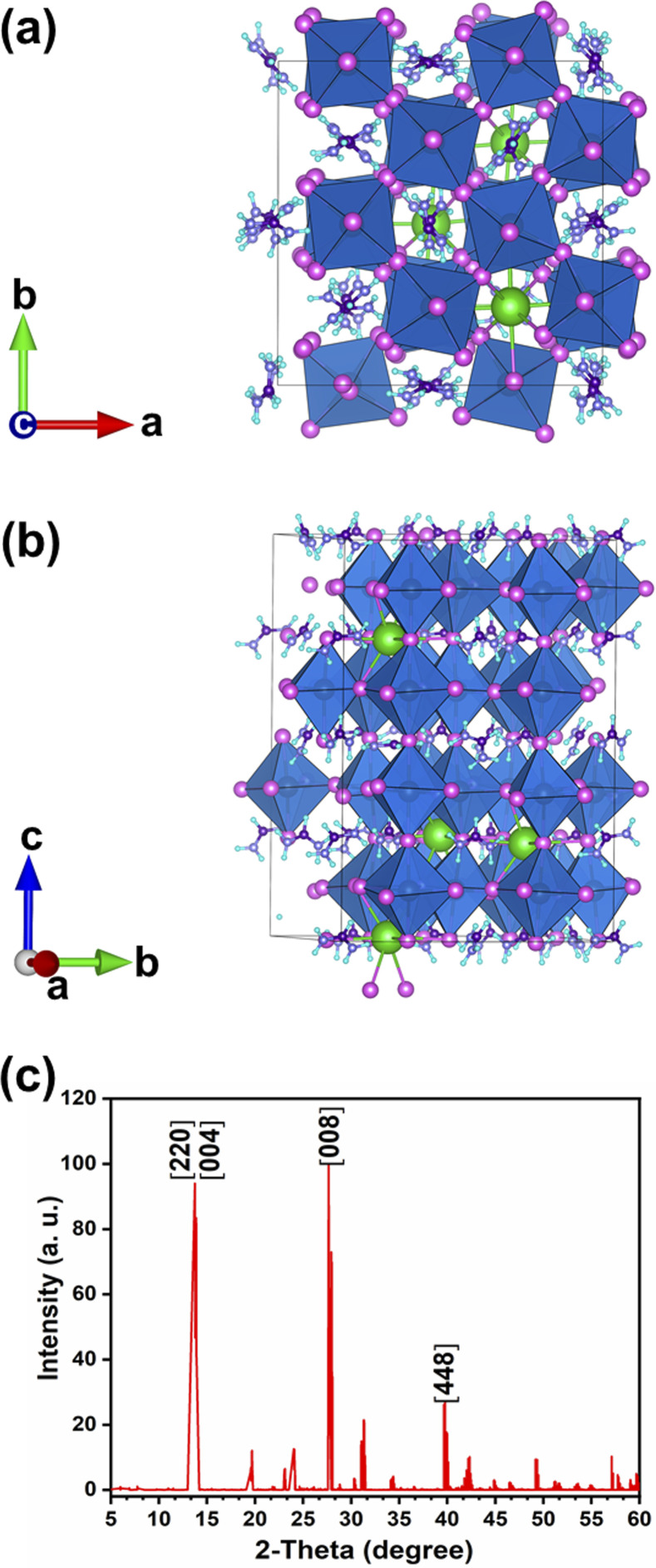
Schematic of the supercell for Cs_0.17_FA_0.87_PbI_3_: (a) top view, and (b) side view. Green, grey, pink, indigo, light-slate blue, and aqua atoms corresponds to Cs, Pb, I, C, N, and H atoms, respectively. (c) The related DFT simulated XRD pattern of Cs_0.17_FA_0.87_PbI_3_.

DFT calculations predict a stable phase for Cs_0.17_FA_0.87_PbI_3_ with a tetragonal unit cell having the dimensions of *a* = 8.93 Å and *c* = 13.01 Å, which are very close to the ones for MAPbI_3_. The average Pb–I bond length, the horizontal, and vertical I–Pb–I angles were calculated to be 3.24 Å, 174°, and 173.6°, respectively. The corresponding standard deviations were 0.045 Å, 2.79°, and 3.0°, respectively. The average vertical and horizontal I–Pb–I bond angles are less than the corresponding values in pure FAPbI_3_ and CsPbI_3_ perovskites, which were calculated *via* DFT with a similar methodology.^18^ The DFT optimized structure of Cs_0.17_FA_0.87_PbI_3_ (Fig. 1a and b) shows a different tilting of PbI_6_ octahedra depending on the surrounding monovalent cations. Here the local and steric effects from the monovalent cation can distort the octahedron affecting the Pb–I bond lengths and the tilting of the octahedron. Similar effects have been observed before in chromium(iii) complexes.^30^ The average octahedra tilting angle was 15.54° while the octahedra next to the Cs cations show strong distortions coming from broken symmetry of surrounding atoms. For instance, tilting angles, vertical Pb–I bond length, and vertical I–Pb–I bond angles as much as 29°, 3.36 Å, and 168° were obtained for highly distorted octahedra next to caesium cations.

Distortion factors of octahedra have been calculated according to eqn (3):3
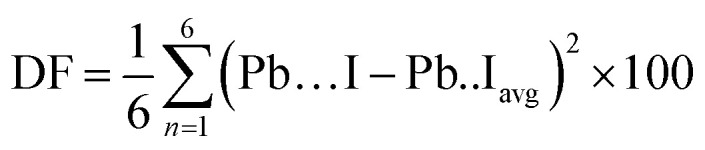


The distortion factors for high-symmetry octahedra surrounded by FA cations and highly distorted octahedra surrounded by mixed-FA-Cs cations were estimated to be 0.025 and 0.64 respectively.

The simulated X-ray diffraction (XRD) pattern is presented in Fig. 1c. Previously, a similar distortion induced by the presence of an extra Rb cation in the Cs_1−*x*_Rb_*x*_PbBr_3_ mixed perovskite was verified experimentally and through DFT calculations.^19^

**Fig. 2:**
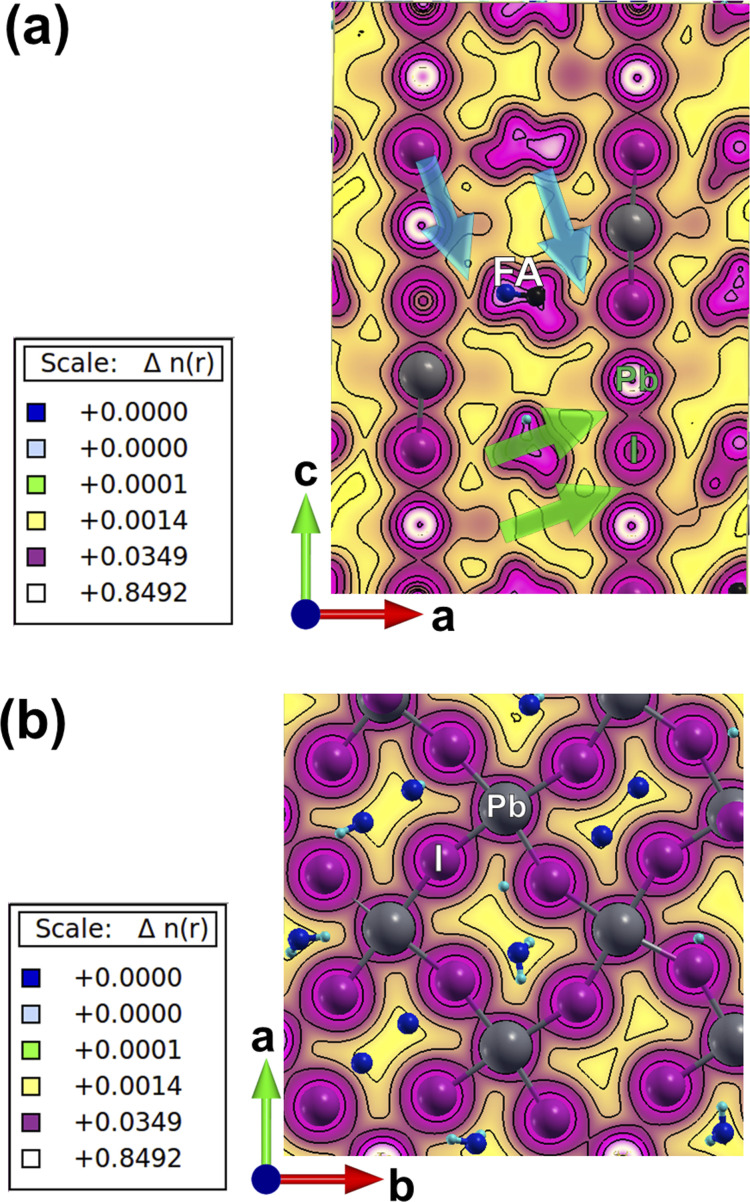
Charge-density plots for Cs_0.17_FA_0.87_PbI_3_: (a) side view and (b) top view. Grey and purple atoms correspond to lead and iodine respectively. Green arrows in (a) show the asymmetric distribution of the electronic cloud in I–Pb–I bond due to the octahedral distortion. Blue arrows in (a) show the interaction of the electronic cloud in between the FA monovalent cation and lattice iodine, and the polarizability induced to the corresponding chemical bond.

**Fig. 3:**
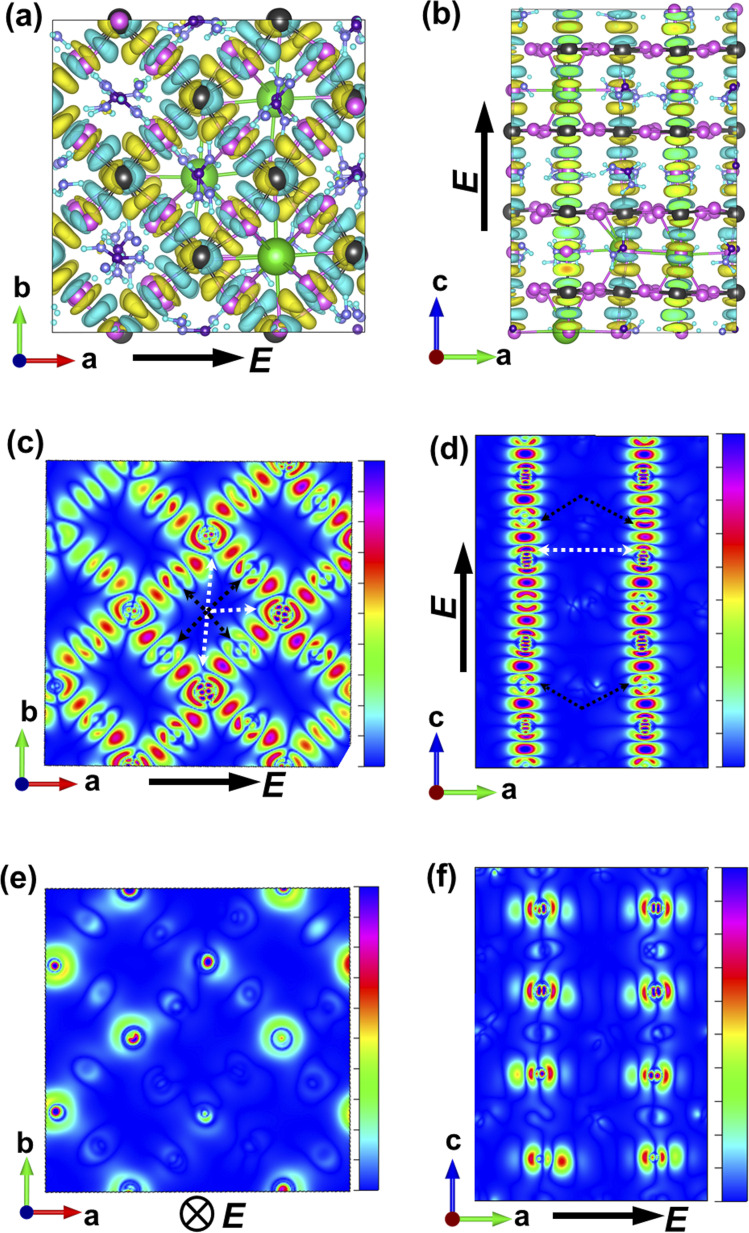
TD-DFT results for the charge-density response. (a) Overall charge-density response with an iso-surface density value of 1.5 × 10^−4^ in the *ab* plane, and (b) the iso-surface density value of 4.5 × 10^−5^ in the bc plane. Polarization of incoming light is depicted in all the figure. (c–f) Charge-density response is plotted in different planes of the lattice: (c and f) light polarization is along the *a*-axis, (e and d) light polarization is along the *c* axis. White and black arrows are respectively pointing towards the lead and iodine orbitals in the lattice highlighting the local polarizabilities in the mixed perovskite lattice.

Charge-density plots show the covalent bond formed between the lead and iodine in the perovskite lattice (Fig. 2). The inorganic network of PbI_6_ octahedra is responsible for charge transport through the covalent chemical bonds and energetically viable path in between the metal and the halogen, as can be extracted from the top view and side view plots (Fig. 2a and b). Due to the distortions induced by the presence of caesium atoms, which are accompanied with higher tilting of the octahedra, there is an asymmetry in between some of the I–Pb–I chemical bonds perpendicular to the crystallographic ab plane (marked with green arrows in Fig. 2a). This asymmetry induces a polarizability in the electronic cloud around the iodine and lead atoms and could have an impact on the charge transport, ion migration, and light absorption along the crystal axis. To further investigate this, we have performed TD-DFT calculations and provide charge-density response plots at the excited state (Fig. 3).

There is a ground-state charge-density overlap in between atomic orbitals for some of the FA molecules and iodine atoms (marked with blue arrows in Fig. 2a) where a polarization is induced on the formed chemical bonds, and a hydrogen bond is formed in between I and H atoms (Fig. 2a). The impact of such an overlap on the charge transport along the crystallographic *c* axis needs to be resolved. Such an induced-polarization in the ground-state charge density of I orbitals is not observed for pure perovskites such as FAPbI_3_ and MAPbI_3_, and probably resulted from a highly distorted local structure originating from the broken symmetry of the lattice due to the presence of mixed monovalent cations. These asymmetries in charge-density distribution in physical space are accompanied with dissimilarities in the partial density of states (PDOS) of electronic levels in energy space, *i.e.* when one compares the partial density of different lead and iodine atoms in the lattice of mixed perovskite (Fig. S2†) and compare it with simple FAPbI_3_ (Fig. S3†). These PDOS features and the corresponding differences in between different lattice atoms are absent in the case of single monovalent cation perovskites (Fig. S2 and S3†).

The charge-density response plots (Fig. 3a and b) show the initial distribution of the electronic cloud in the conduction band (CB) after excitation and before the lattice relaxation. The charge density response has the same overall symmetry of the lattice of Cs_0.17_FA_0.87_PbI_3_ considering the charge excitation from the iodine's 5p to the lead's 6p orbitals with respect to the polarization of the incoming light (Fig. 3a and b) and in agreement with pure FAPbI_3_.^29^ Plotting the profiles of the excited-charge density within special planes in the lattice, *i.e.* the plane which includes the centre of mass of lead and iodine, reveals the asymmetry and polarizabilities of the electronic cloud (marked in Fig. 3c–f) which are absent in pure FAPbI_3_ and will be further discussed below.

### Electronic structure

**Fig. 4:**
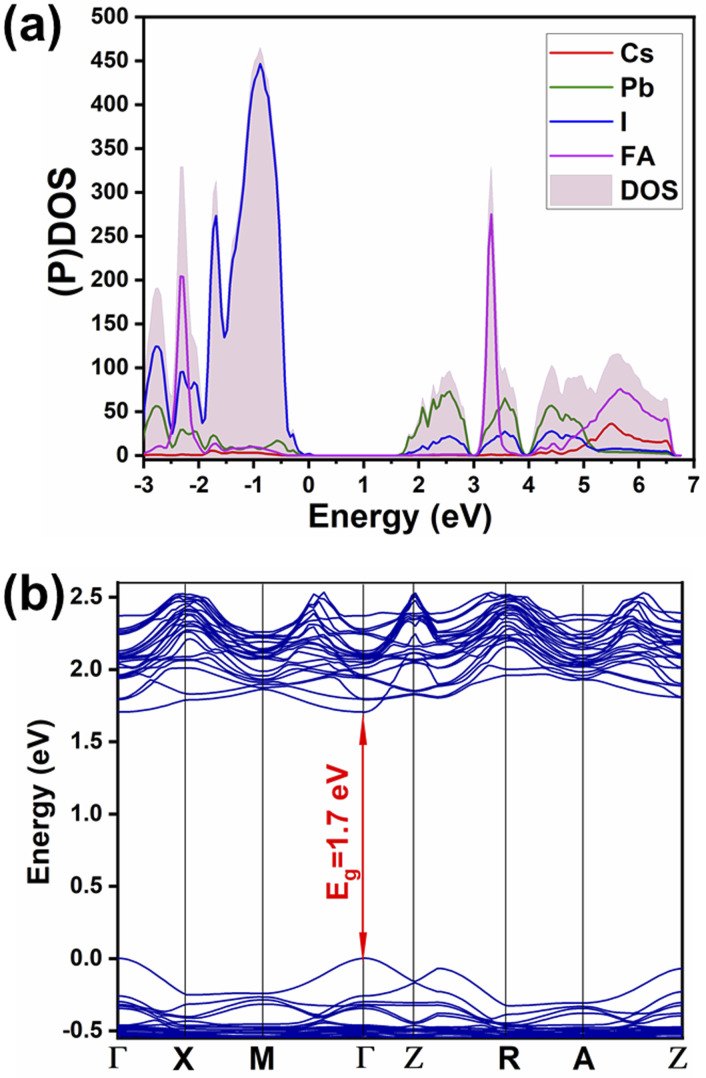
(a) Partial and total density of states ((P)DOS and DOS), (b) band structure of Cs_0.17_FA_0.87_PbI_3_.

Partial density of states (shown in Fig. 4a) evinces the contribution of mainly 6p orbitals from lead (partially hybridized with I) to the CB minimum, and 5p orbitals from iodine (partially hybridized with Pb) mainly to the maximum of the valence band (VB), in agreement with previous reports for MAPbI_3_. There is no contribution from monovalent cations FA and Cs in the vicinity of the CB minimum and of the VB maximum. Hence the main physical phenomena, such as charge transport and excitation, happen in the inorganic network. Therefore, there is no meaningful difference between the mixed-cation perovskite and FAPbI_3_ in terms of orbital contributions in density of states. Furthermore, from the PDOS spectrum, the possibility of charge excitation from iodine to the FA cation can be evidenced when using a violet photon (*i.e.*, 3.25 eV) illumination that agrees with our previous reports.^19,29^ The main difference is in the local density of states of atoms where different atoms within the lattice experience different surroundings and different local distortions of PbI_6_ octahedra. Therefore, a difference in partial density of states in the energy space can be noted, *i.e.*, the differences depicted in Fig. S2† for the p orbitals of different lead and iodine atoms in mixed perovskite lattice. Similar atoms in the pure FAPbI_3_ perovskite show no difference in the partial density of states (Fig. S3†).

The calculated band structure of Cs_0.17_FA_0.87_PbI_3_ (Fig. 4b) indicates a direct band gap of 1.7 eV at Gamma point and low effective masses for electrons and holes, convenient for solar light absorption and charge transport in solar cell applications. However, charge transport is not symmetric in the ab plane and, in special directions within the ab plane, one can find high effective masses for holes, *i.e.*, directions *X* → *M* and *R* → *A* (Fig. 4b). Here, effective masses of electrons and holes were calculated based on band theory in the independent electron approximation (eqn (4))^24,31^4
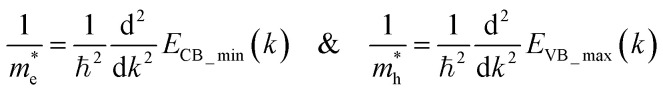
where 
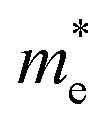
 and 
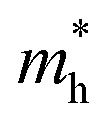
 are the effective masses of the electrons and holes respectively, *ħ* is the reduced Planck constant, *k* is the reciprocal lattice vector and *E*_CB_min_ and *E*_VB_max_ are representative of the minimum and maximum of the energy at the CB and VB, respectively and the corresponding values are reported in Table 1.

**Table 1:** Effective mass of charges for Cs_0.17_FA_0.87_PbI_3_ at different reciprocal paths in terms of the free electron mass *m*_0_

	*Γ* → *X*		*X* → *M*		*Γ* → *Z*	
Structure	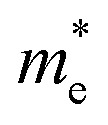	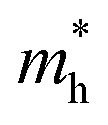	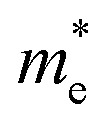	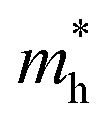	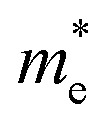	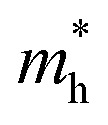
Cs_0.17_FA_0.87_PbI_3_	1.1	0.61	1.4	7.07	0.04	0.25
CsFAPbI_3_	0.094	0.15	6.3	0.43	0.25	0.05

In polycrystalline films with random orientation, the effective mass can be taken as an average scalar value, while for anisotropic crystal structures the effective mass is a tensor that depends on the direction of the charge carriers in reciprocal space. The calculated effective masses of electrons and holes for *Γ* → *X*, *X* → *M*, and *Γ* → *Z* are reported in Table 1, where for example *Γ* → *Z* effective masses for the electron and the hole are 0.04 *m*_0_ and 0.25 *m*_0_, respectively; *m*_0_ is the mass of the free electron.

Here one can note that he effective mass in the *X*-direction is significantly increased in the mixed-cation situation compared to the case with the FA cation, with more than 10 time increase in the electron effective mass and 4 times increase in the effective hole mass from 0.094 *m*_0_ and 0.15 *m*_0_ to 1.1 *m*_0_ and 0.61 *m*_0_ using a mixed-cation material. This can be contrasted to the situation for the charge carrier in the *Γ*–*Z*-direction with a significant lowering of the electron effective mass from 0.25 *m*_0_ to 0.04 *m*_0_, while keeping the effective hole mass at or below 0.25 *m*_0_ (Table 1). As the reduced mass (*μ*) is closest to the lightest mass, as evident from the well-known formula 
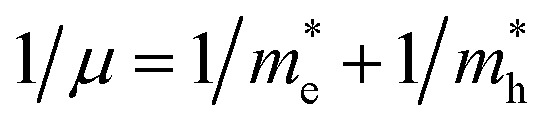
, this implies a substantial lowering of the charge mobility in the *Γ*–*Z* direction. From these results, orientation of crystals in the *Γ*–*Z* direction in-between charge collecting electrodes will thus be highly preferred in comparison to a *Γ*–*X* orientation in Cs/FA mixed lead-halide perovskites but, on the other hand, an increased transfer in the preferential direction.

## Discussion

While MAPbI_3_ based PSCs encounter volatility and instabilities, FAPbI_3_ PSCs have much higher stabilities both in device efficiency and material composition. As mentioned, the photovoltaic active phase of FAPbI_3_ can be stabilized with the addition of caesium atoms in the perovskite lattice. In contrast to FAPbI_3_, the presence of caesium atoms in CsFAPbI_3_ stabilizes the room temperature phase structure (referred to as the black phase) and prevents the formation of the non-photovoltaic phase (yellow phase) of FAPbI_3_. Different strategies for improving phase stability has been discussed extensively in the literature,^32^ with the most appreciated strategy is the addition of caesium atoms to the FAPbI_3_ lattice, thus making Cs_*x*_FA_1−*x*_PbI_3_ one of the most convenient and stable perovskite solar cell materials in this perovskite class. A comparison between the Cs_0.17_FA_0.87_PbI_3_ and pure FAPbI_3_ shows that there are negligible differences in the main electronic and structural properties such as the band gap, the charge-density distributions, the partial and total density of states; and the main atomic orbitals responsible for the carrier creation and transport are similar, leading to high device efficiencies for both cases (See ESI).† Differences are instead seen in band structure, effective mass of carriers, and features in the PDOS spectrum away from band edges that are important for photovoltaic performance of the majority of light with wavelengths not matching the lowest band gap transition. Compared to FAPbI_3_, however, the differences in effective masses of Cs_0.17_FA_0.87_PbI_3_ (Table 1) modify the electronic transport and its directional dependence which ultimately affect the overall fill factor of the corresponding device. Furthermore, as mentioned, in Cs_0.17_FA_0.87_PbI_3_ there are asymmetries in the chemical bonds (marked in Fig. 2 and 3) in between the Pb–I that originate from the lattice distortions induced by the broken symmetry due to presence of Cs atoms. These monovalent-cation-induced asymmetries cause a non-uniform polarization which can have impacts on the physical properties. The uneven distribution of polarization induced by the incoming light (as is clear from the distribution of the electronic cloud in lead p_*z*_ orbitals after excitations: Fig. 3e and f) is absent for the MAPbI_3_, FAPbI_3_, and CsPbI_3_ perovskites from our previous reports^29^ Such a polarization variation immediately after the excitation can have a meaningful impact on the amplitude of electron–phonon interactions^33^ and therefore in the rate of electron cooling of the mixed-cation perovskite according to the following equations. The electron–phonon, or more generally, electron polarization interaction Hamiltonian can be described by:

5 *H*′ = ∫*D⃑*(*r* − *r*′)·*P⃑*(*r*)d*r*

where *P⃑* is the polarization, *r* and *r*′ represent the location operator, and *D⃑* is the field produced at *r* due to an electron present at *r*′.6
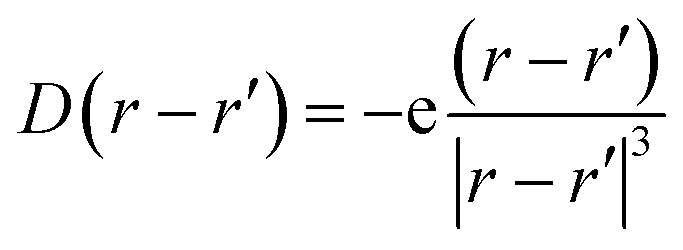


This interaction Hamiltonian has an explicit polarizability dependence and in special cases is equivalent to the bulk Fröhlich Hamiltonian.^33^ For this study, one can account for the polarization created by oscillating ions which has two parts, one from the ions and one from the electronic cloud:7
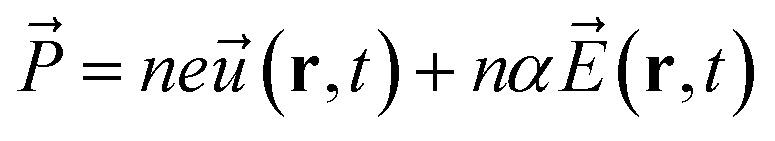
here *P⃑*, *n*, *e*, *α*, and *E⃑* are respectively the film polarization, the number of ion pairs in the unit cell, the magnitude of the partial charge on the lattice ions, the polarizability from the electronic cloud, and the external electric field. In our study, for the Pb–I oscillations in inorganic network, *u⃑* can be defined as the displacement in between the Pb and I atoms:8
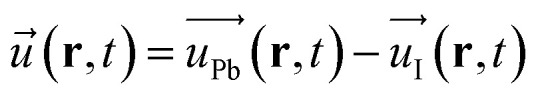
where **r**, *t* are the location vector and the time, respectively.

The local polarizability variations, originating from both local geometrical factors and the electronic cloud distributions, can affect and be affected by the time dependent atomic scale motion of the ions.^33^ Therefore, both asymmetry ionic displacement and uneven charge-density distributions can affect the electron–phonon interaction amplitude based on the equations. Immediately after the excitation and the thermalization, the excess energy of hot electrons is transferred to the lattice *via* electron–phonon interactions. Therefore, the amplitude of electron–phonon interactions (either acoustic or optical phonons) together with other factors, such as the density of available phononic states, are related to the cooling rate and the excited state lifetime of the hot carriers. As a consequence, the above-mentioned polarisation differences can also affect the excited state lifetime of hot carriers.^34^ In accordance with this, a higher excited-state lifetime of carriers for mixed-cation perovskites compared to pure perovskites has been experimentally reported by Brauer *et al.*^11^ However, they did not consider the roles of electron–phonon interactions in their conclusions, which we here find to be important to understand the origin of the effect.

Monovalent cation induced polarizabilities can further affect the light absorption selection rules and directional dependence of the dielectric constant at steady state.^35^ This phenomena is also present in pure cubic FAPbI_3_ due to the lattice asymmetry but to a much smaller extent.^35^ The local differences in the atomic scale electronic cloud distribution which can be translated to differences in directional light absorption, electron–phonon interactions, and even in the dielectric constant, can be considered as the main factor responsible for many of the observed differences in the physical behaviour of mixed-cation perovskites, when one compares it to single monovalent cation perovskite materials.

In addition, the broken symmetry observed in this study, which is evident in the location and distribution of the electronic cloud in the excited and ground states (Fig. 2 and 3) and in the differences in partial density of states of different iodine atomic orbitals (Fig. S3†) is important to consider. This can affect the barriers of ionic movement in the lattice (in agreement with a recent report^15^) and also contribute to routes for ionic movement, or acting as an impediment for it. With further experimental verifications, this can potentially be considered as one of the main candidate reasons behind the superior device stability and less observed hysteresis in mixed-cation perovskite solar cells. Further calculations with coupled experiments can draw a more comprehensive picture for these above-mentioned factors, *i.e.*, calculations of barriers of ionic movement and studying the phonon–electron interactions or directional dependence of dielectric constant in comparison with single crystal experimental studies. These however are beyond the scope of the current study and only suggested here as future endeavours.

According to our results, asymmetry plays an important role in improving the performance of solar cells as outlined in the above experimental and theoretical works. For example, based on a joint theoretical/experimental study^15^ of different mixed cation perovskites, the level of impeding iodine ions movement can be at the level of 12% to 78%. The trend for reported values has been explained based on the direct impact of cation size mismatch which is directly translated to lattice distortions and asymmetries as we emphasized. Moreover, the slow component of excited state carrier lifetime of mixed perovskite has been reported one order of magnitude higher than pure perovskite.^11^ The higher population of higher excited state levels and different symmetry-based selection rules demonstrated is an important factor for this, emphasizing the role of asymmetry in the hot carrier lifetimes in perovskite solar cell materials. The same role-play of symmetry-based selection rules are used for dielectric constant and light absorption^35^ and is anticipated to play an analogous role in the context for the above analysed phenomena. The possible impacts of the DFT-calculated parameters on the device performance parameters are summarized in Table S2 of ESI.†

## Conclusions

In summary, we have investigated and reported the electronic structure of Cs_0.17_FA_0.87_PbI_3_ for solar cell applications, one of the main compositions with a band gap of 1.7 eV. It has a band gap of interest for inverted perovskite solar cell devices for tandem application in combination with Si or other semiconductors with a band gap around 1.1 eV such as Copper Indium Selenide, CIS, or low Ga content Copper Indium Gallium dis-Selenide, CIGS. Our results show that the simple optoelectronic descriptors such as band gap and charge density of Cs_0.17_FA_0.87_PbI_3_ differs negligibly from that of a pure monovalent cation-based perovskite, while local symmetry, band dispersion, and excited state polarizabilities are significantly different. In particular, the orientational disorder and lattice distortion induced by mixed cations in the lattice gives rise to polarizabilities in the charge density of both the ground and excited states before the thermalization/lattice relaxation occurs. The impact of such polarization and distortions on the electron–phonon interactions, on the lattice relaxation, and on the excited-state lifetime of hot electrons are different for the mixed A-site cation system compared to single A-site cation perovskites. The results presented herein show that, although the main electronic structure of monovalent and mixed-cation perovskites is similar, the asymmetry and distortion-induced charge polarizabilities in the lattice of mixed-cation perovskites should have strong impacts on the photovoltaic performance at operational conditions. The same conclusions can be extended for mixed perovskites with different compositions in general and shed light for further understanding of higher performance and stabilities in mixed-cation-based perovskite solar cell devices.

## Author contributions

R. I. performed the majority of the quantum mechanical calculations, the initial data analysis, and wrote the first draft of the manuscript. C. B., M. P., and T. E. performed additional analysis, interpretations, and writing of the manuscript. M. P. and T. E. supervised and guided the work.

## Conflicts of interest

There are no conflicts to declare.

## Supplementary Material

RA-012-D2RA04513C-s001
