# Supplementary material for: Excited-state charge polarization and electronic structure of mixed-cation halide perovskites: the role of mixed inorganic–organic cations in CsFAPbI3
Source: RSC Adv. 2022 Sep 7;12(39):25415–23. doi: 10.1039/d2ra04513c (PMC9450942; doi:10.1039/d2ra04513c)
Supplement: RA-012-D2RA04513C-s001 [file RA-012-D2RA04513C-s001.pdf]

Electronic Supplementary Material (ESI) for RSC Advances.
This journal is © The Royal Society of Chemistry 2022

Excited-state charge polarization and electronic structure of mixed-cation halide perovskites: The role of mixed inorganic-organic cations in $CsFAPbI_3$.

Roghayeh Imani,[a] Carlos H. Borca[b], Meysam Pazoki*[c,d], and Tomas Edvinsson *[a]

Supplementary Information

[a.] *Department of Materials Science and Engineering, Solid State Physics, Ångström Laboratory, Uppsala University, Box 34, 75121 Uppsala, Sweden.*
[b.] *Department of Chemical and Biological Engineering, School of Engineering and Applied Science, Princeton University, Princeton, New Jersey 08544, United States.*
[c] *Institute for Photovoltaics, Stuttgart University, 70569 Stuttgart, Germany*
[d.] *Department of Physics, Shiraz University, Shiraz 71454, Iran.*
Electronic Supplementary Information (ESI) available: See DOI: 10.1039/x0xx00000x

Table S1. Total energy, band gap and total energy per unit cell for supercells consisting of different configurations of Cs/FA monovalent cations inside the supercell.

Structure	Bandgap (eV)	total energy (Ry)	Total Energy (eV)	Energy per unit cell (eV)
1_1	1.70	-11116.122403	-151179.2646	-4724.352
1_2	1.71	-11116.130571	-151179.3757	-4724.355
1_3	1.71	-11116.131741	-151179.3916	-4724.355
1_4	1.71	-11116.164076	-151179.8314	-4724.369
1_5	1.71	-11116.129746	-151179.3645	-4724.355
2_1	1.71	-11116.133767	-151179.4192	-4724.356
2_2	1.71	-11116.126300	-151179.3176	-4724.353
2_3	1.70	-11116.180776	-151180.0585	-4724.376
2_4	1.71	-11116.134188	-151179.4249	-4724.357
3_1	1.71	-11116.134551	-151179.4299	-4724.357
3_2	1.72	-11116.134237	-151179.4256	-4724.357
4_2	1.70	-11116.126110	-151179.3151	-4724.353
6_1	1.70	-11116.132041	-151179.3957	-4724.356
7_1	1.70	-11116.199515	-151180.3134	-4724.384
10_1	1.72	-11116.137569	-151179.4709	-4724.358

Structure 1_1

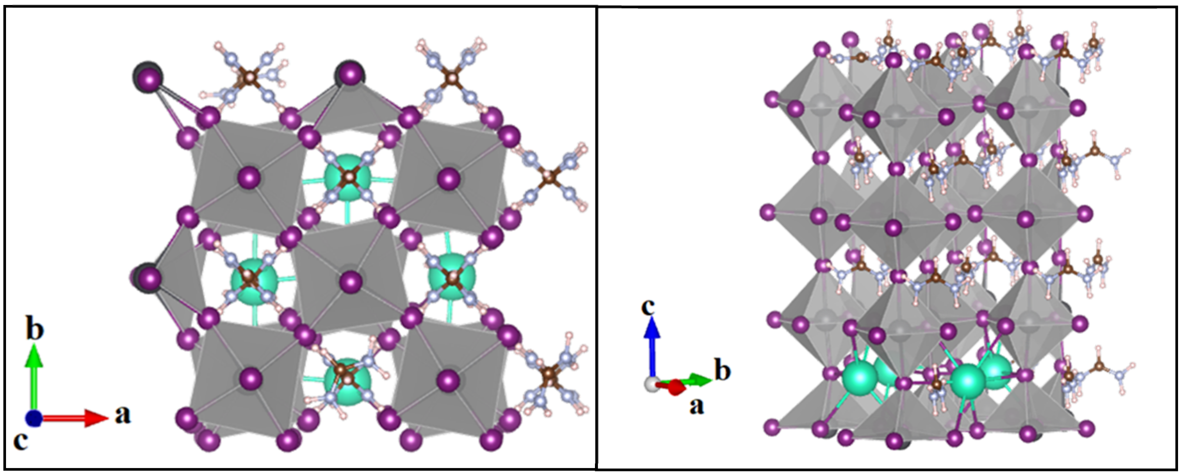

Structure 1_2

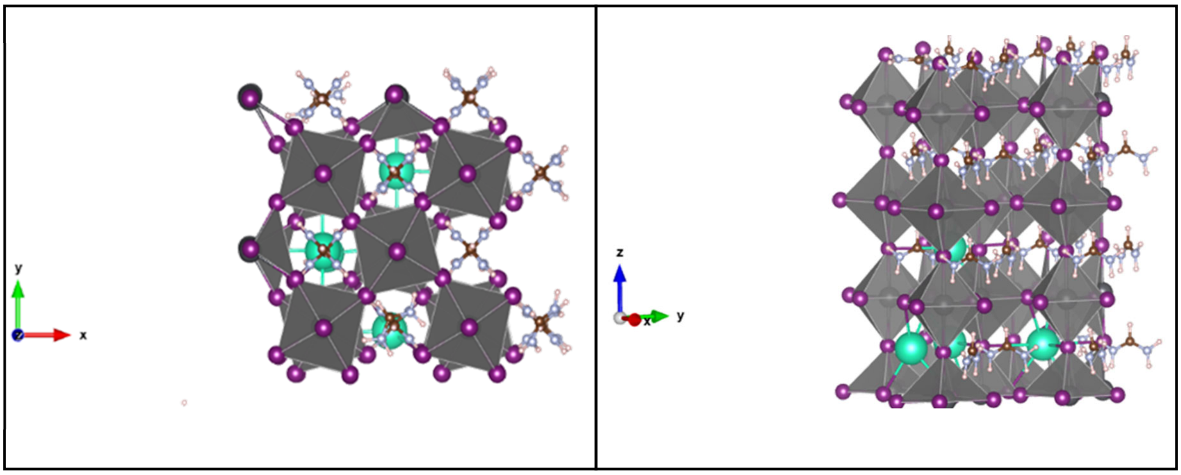

Structure 1_3

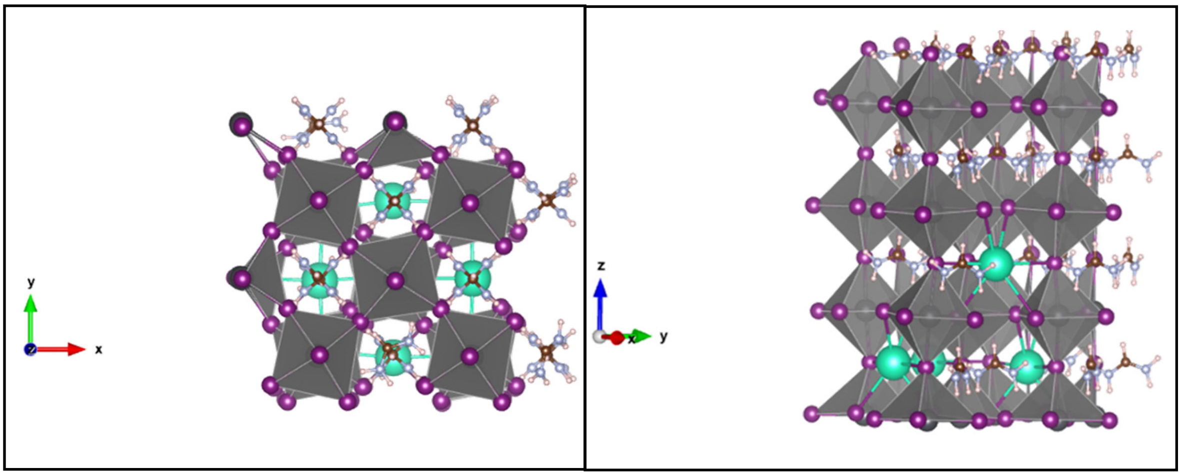

Structure 1_4

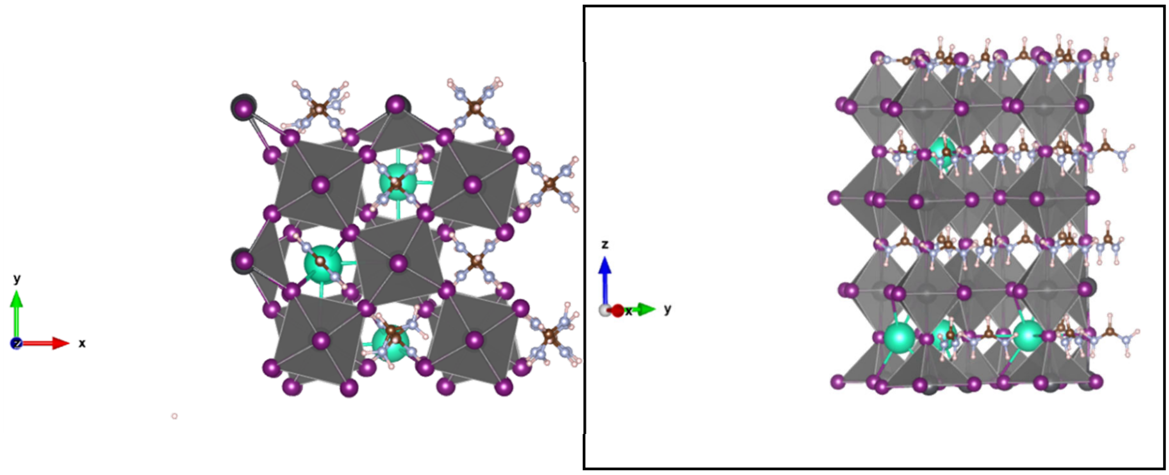

Structure 1_5

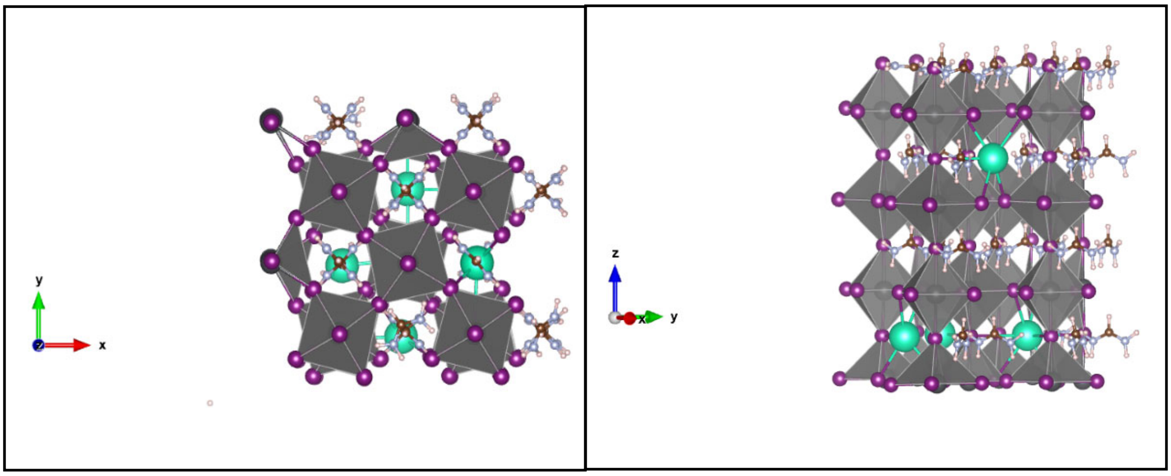

Structure 2_1

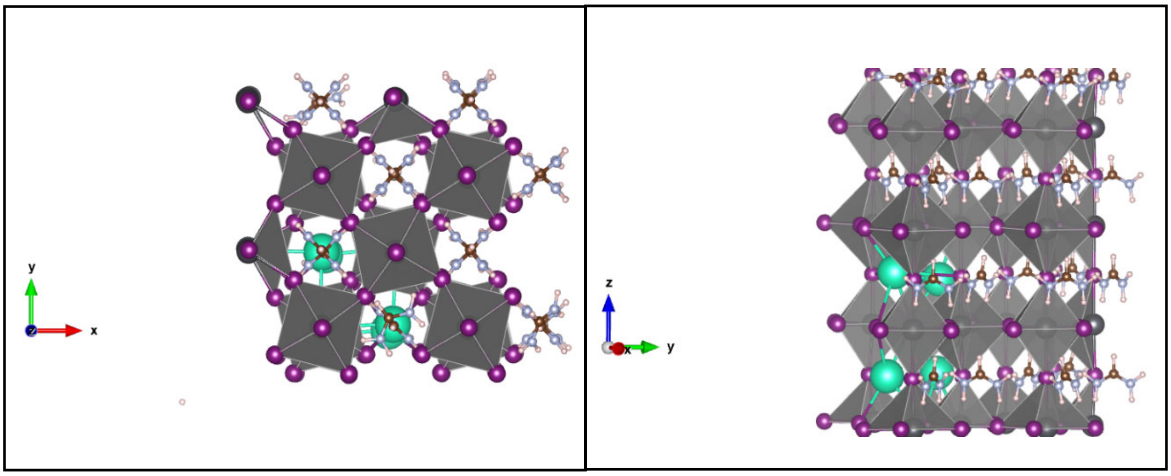

Structure 2_2

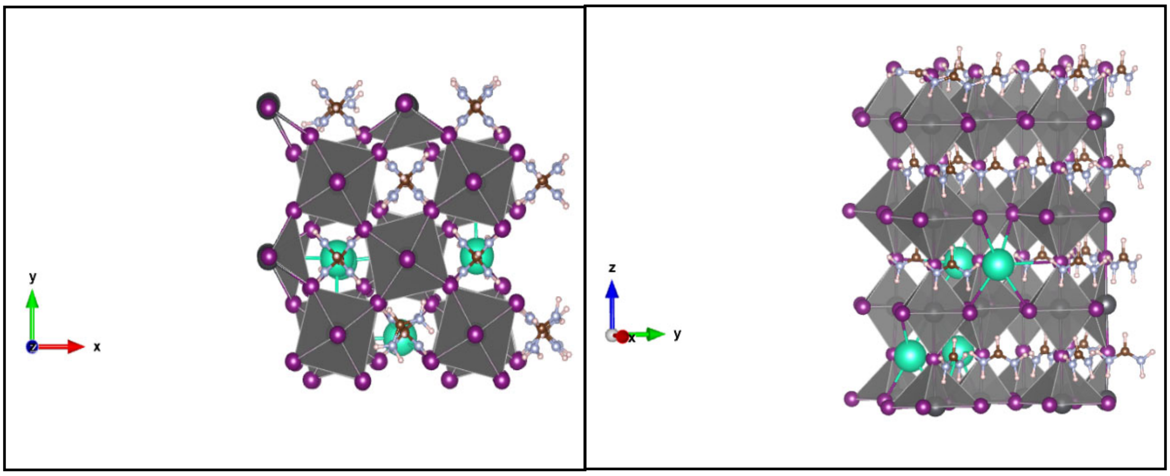

Structure 2_3

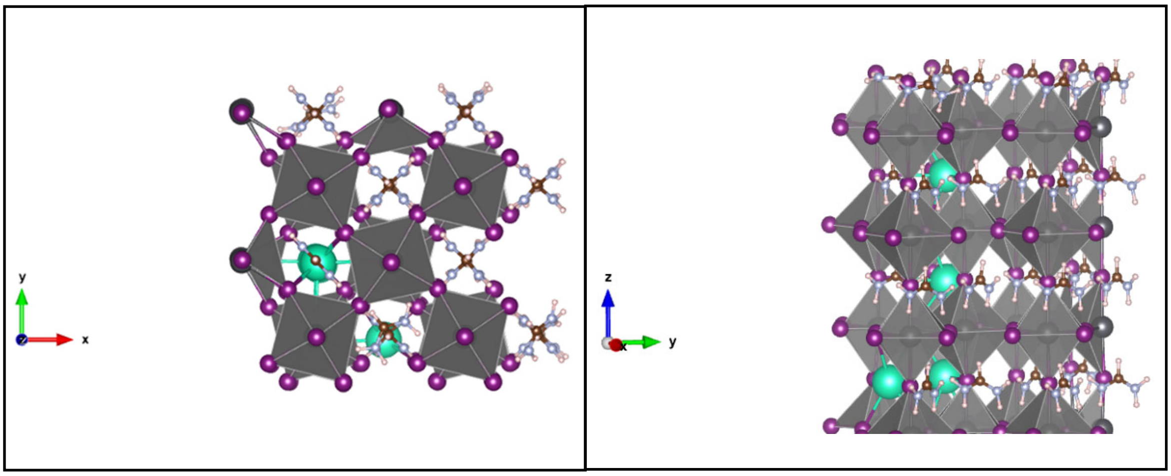

Structure 2_4

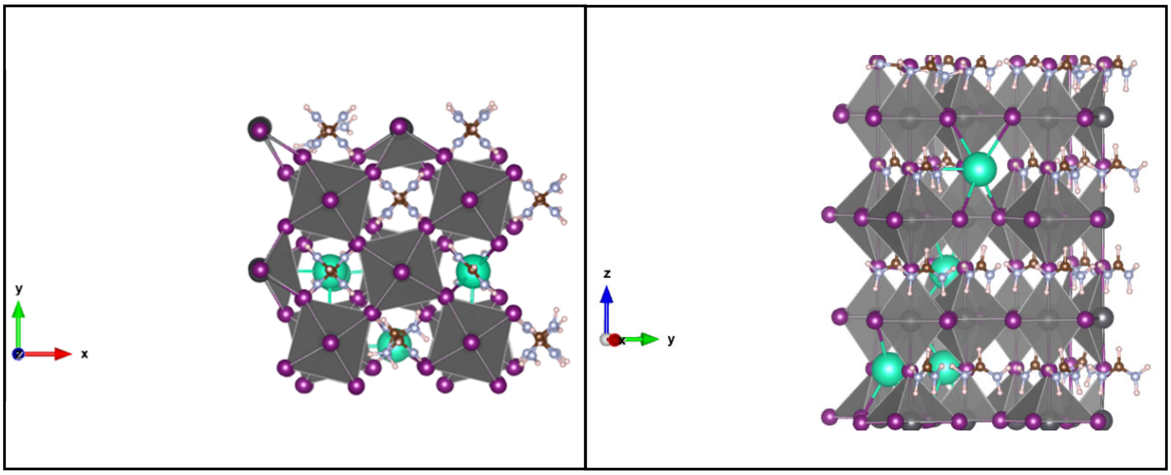

Structure 3_1

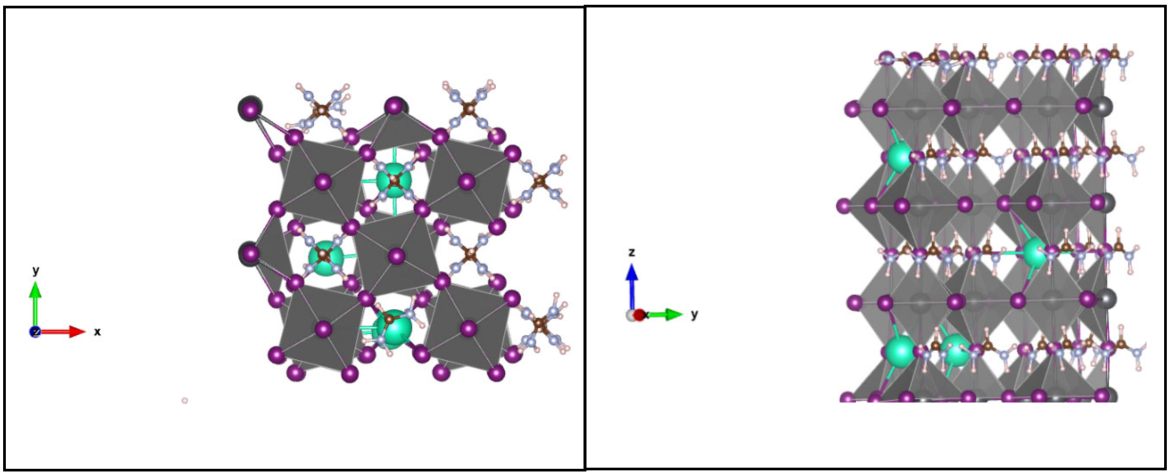

Structure 3_2

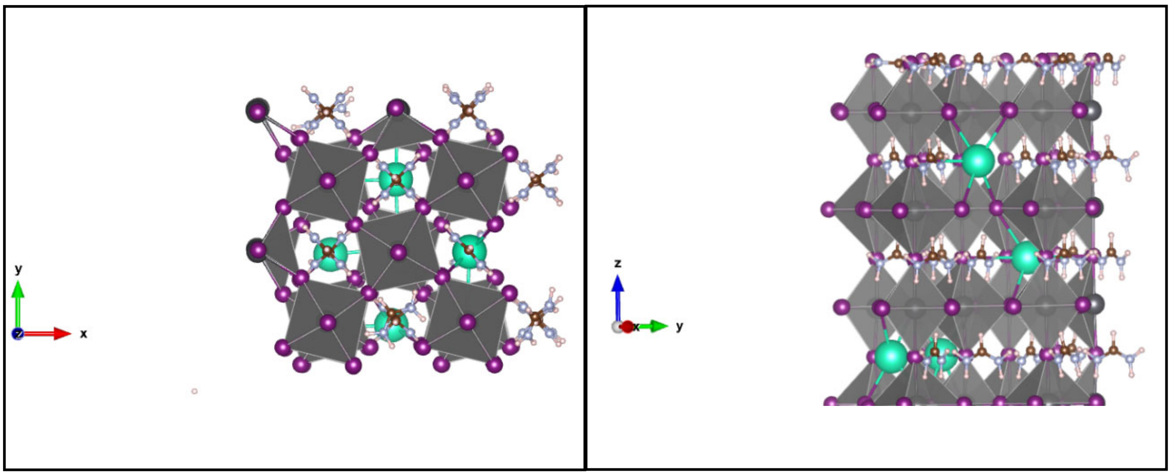

Structure 4_2

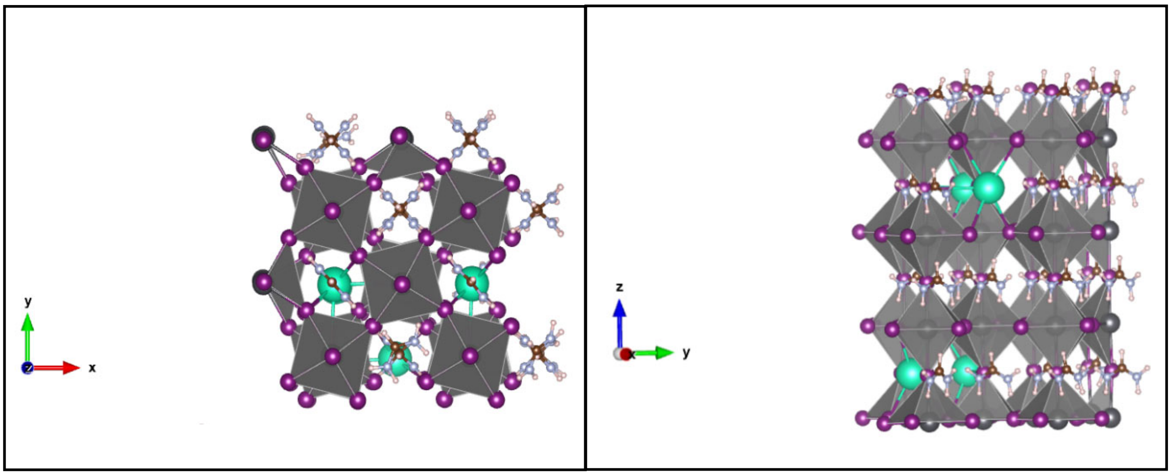

Structure 6_1

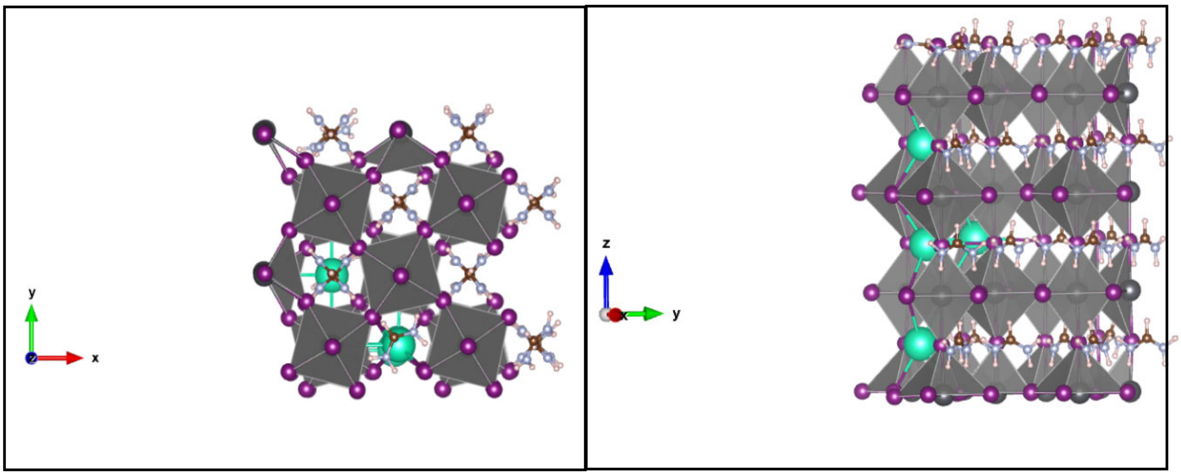

Structure 10_1

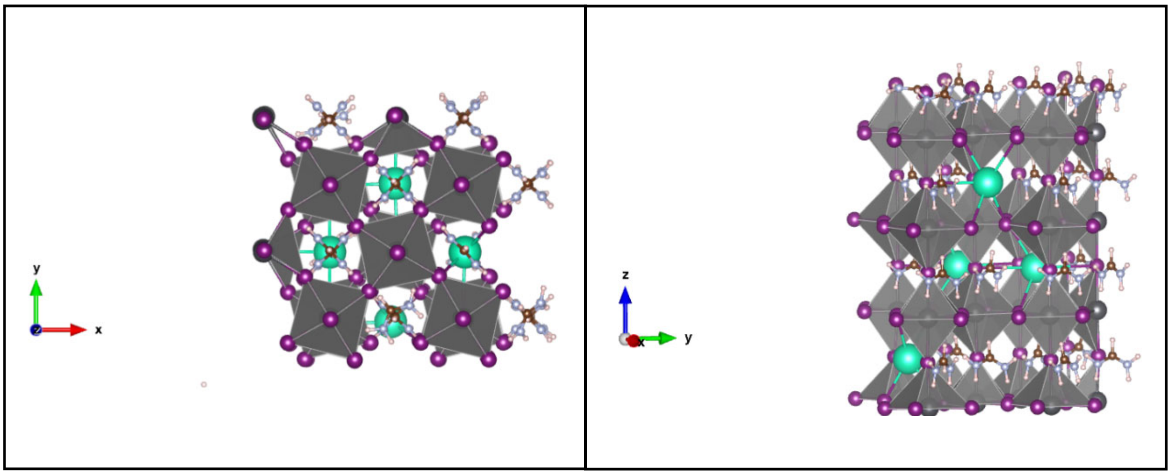

Fig. S1 Schematics of different structures (before relaxation) are presented here. Cyan, Purple, Brown, Blue and White atoms correspond to Cs, Pb, C, N and H atoms respectively. The supercells are visualized inside view (right) and top views(left).

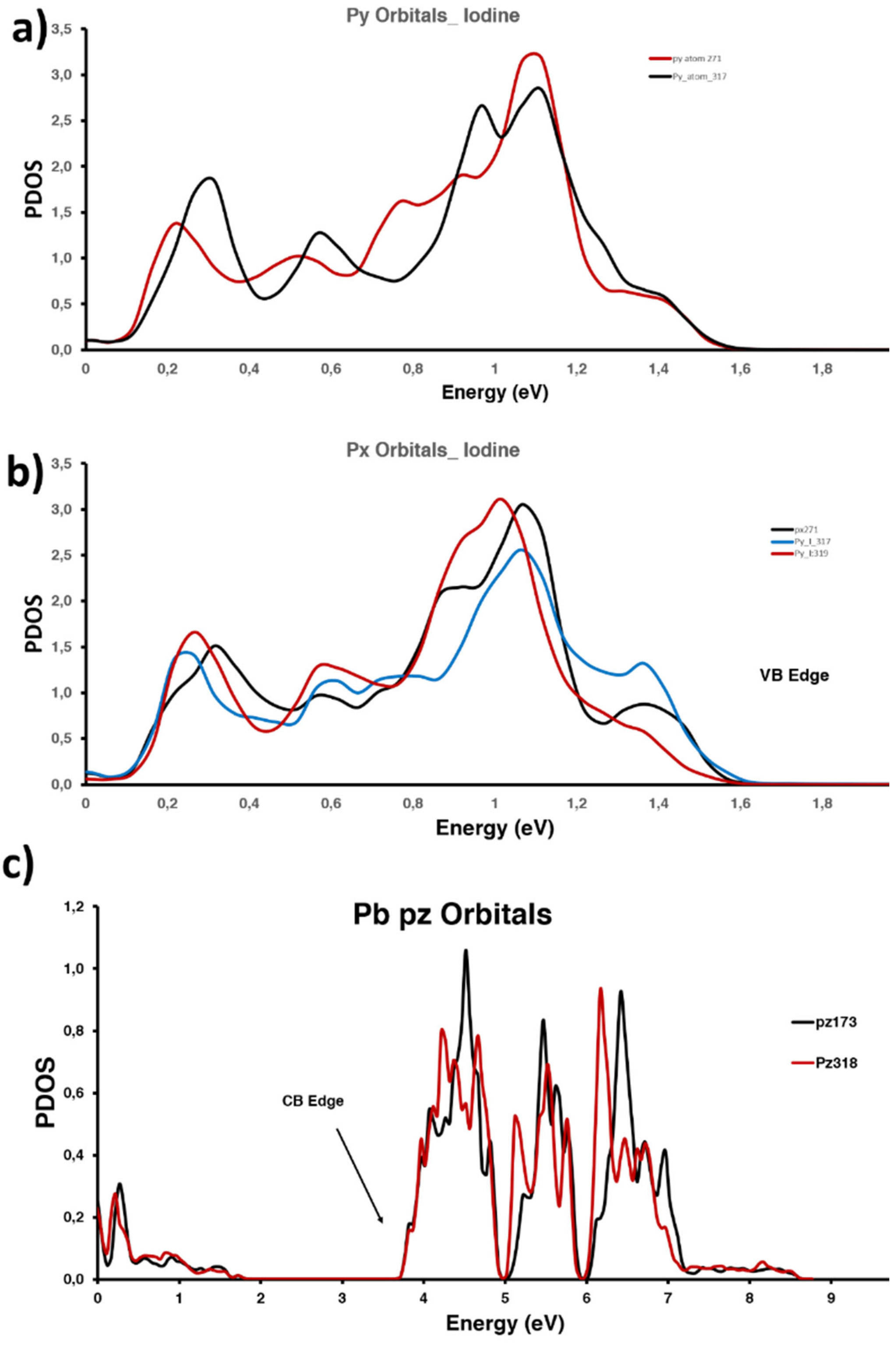

Fig. S2 Partial density of states for **(a)** Py orbitals, **(b)** Px orbitals of different iodine atoms (marked in Figure S4), and **(c)** Pz orbitals of different lead atoms in the lattice. Valence and conduction band edges are marked in the figure.

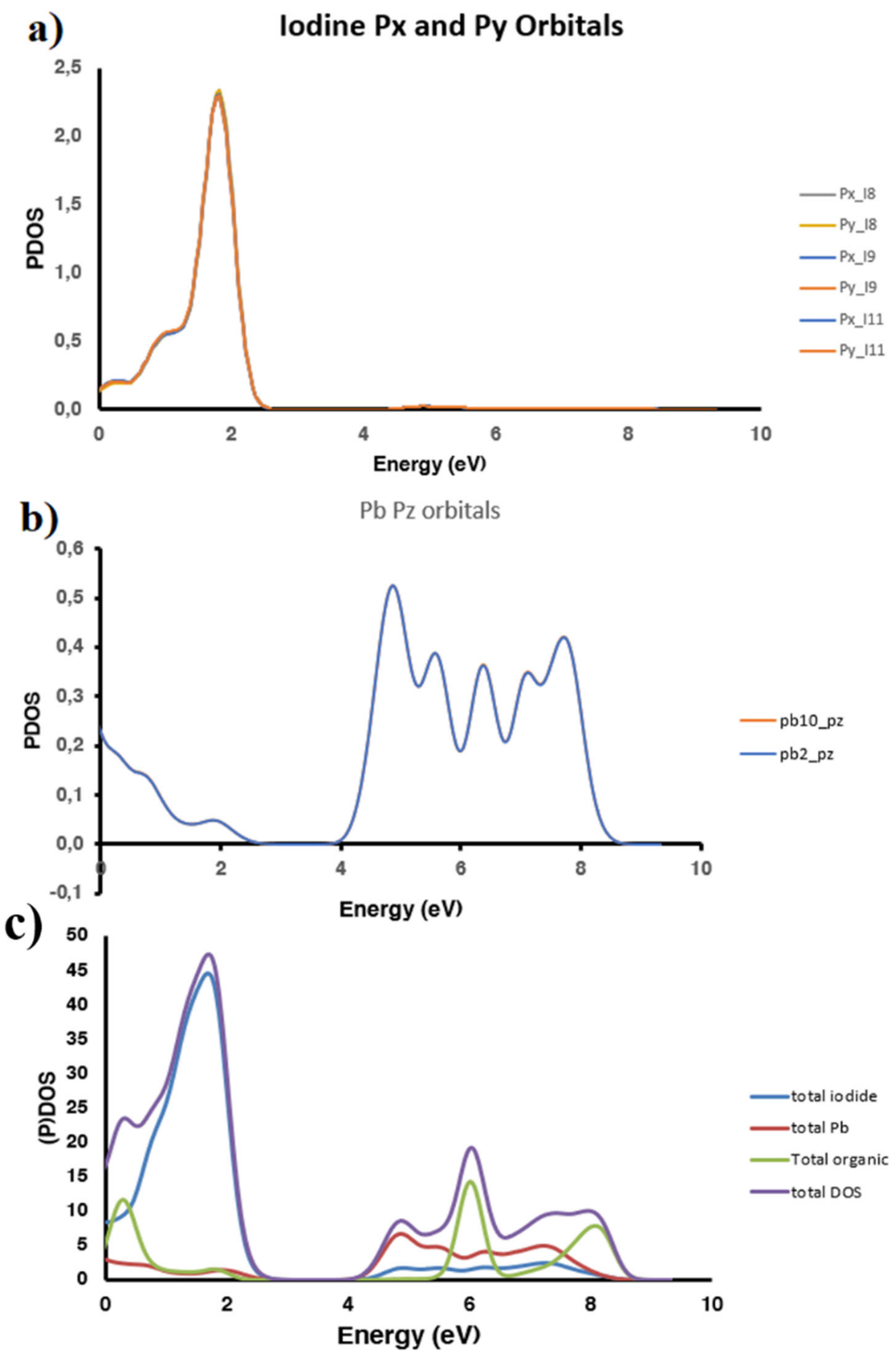

Fig. S3 Partial density of states for **(a)** iodine atoms in Bulk $FAPbI_3$ Px and Py orbitals, **(b)** lead atoms Pz orbitals, and **(c)** the corresponding total DOS. The corresponding atoms are marked in Figure S4.

.

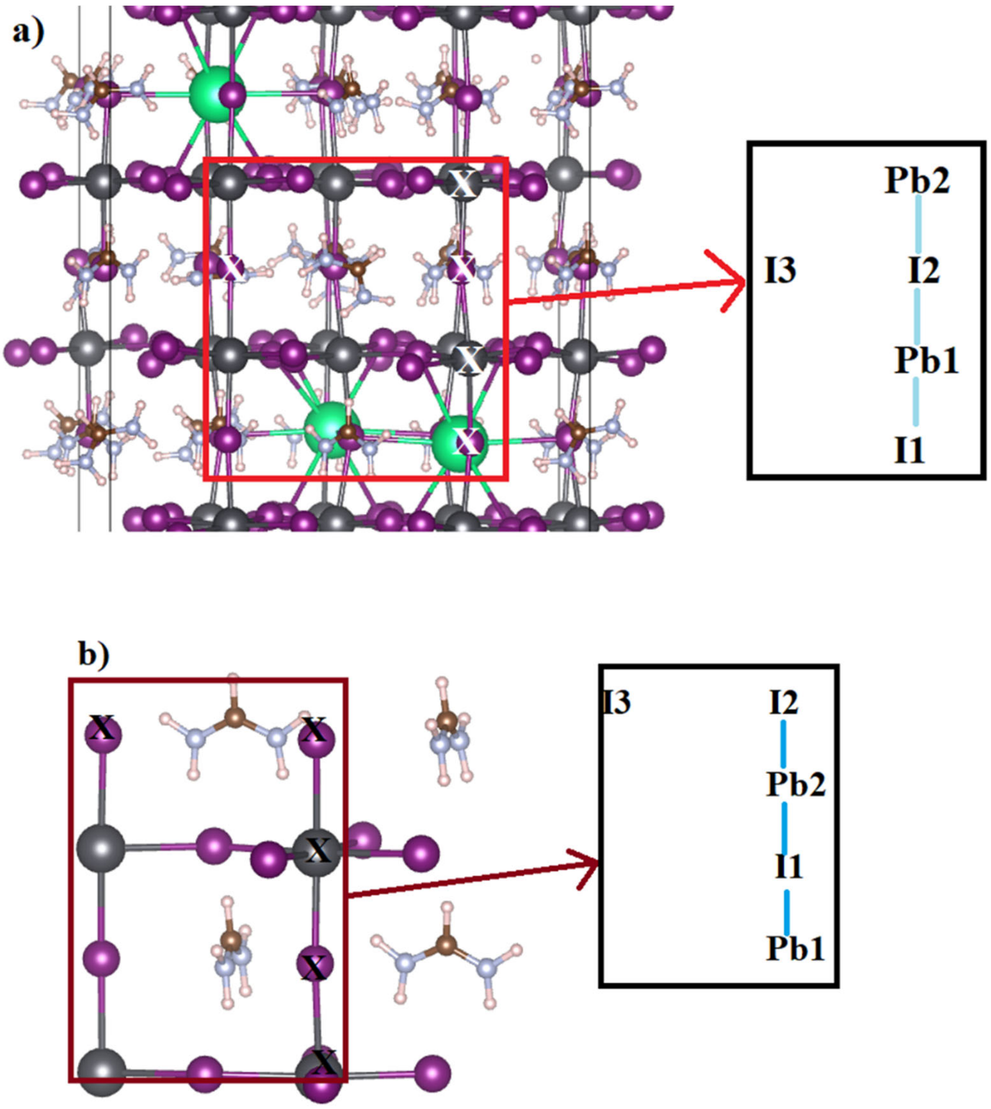

Fig. S4 Labelling the atoms in the lattice of **(a)** mixed $CsFAPbI_3$ and **(b)** bulk $FAPbI_3$ perovskites. The same labelling is used for Figures S2 and S3

Table S2: Comparison of DFT-calculated parameters for mixed cation perovskites compared to monovalent cation perovskites and their impact on device performance

Parameter/Quantity	Percentage of difference	Possible impact on device performance
Effective mass of carriers	400-1400% (current study)	Faster transport time, higher fill factor (FF)
Energy barriers of ionic movement	12-78% [1]	Lower hysteresis, higher stability, higher FF
Excited state lifetime of carriers	One order of magnitude lower [2]	higher possible hot carrier lifetime, higher current, PCE and FF
Light absorption	Not quantified [3]	Higher Jsc and PCE

References

1 D. W. Ferdani, S. R. Pering, D. Ghosh, P. Kubiak, A. B. Walker, S. E. Lewis, A. L. Johnson, P. J. Baker, M. S. Islam and P. J. Cameron, *Energy Environ. Sci.*, 2019, **12**, 2264–2272.

2 J. C. Brauer, D. Tsokkou, S. Sanchez, N. Droseros, B. Roose, E. Mosconi, X. Hua, M. Stolterfoht, D. Neher, U. Steiner, F. De Angelis, A. Abate and N. Banerji, *J. Chem. Phys.*, 2020, **152**, 104703–9.

3 M. Kato, T. Fujiseki, T. Miyadera, T. Sugita, S. Fujimoto, M. Tamakoshi, M. Chikamatsu and H. Fujiwara, *J. Appl. Phys.*, 2017, **121**, 115501–13.
